# Lithium induced reversible Splenial lesion in neuroleptic malignant syndrome like symptoms: two case reports

**DOI:** 10.1186/s12883-020-01742-z

**Published:** 2020-04-30

**Authors:** Han Uk Ryu, Ji Yeon Chung, Byoung-Soo Shin, Hyun Goo Kang

**Affiliations:** 1 Department of Neurology, Jeonbuk National University Medical School, Jeonju, South Korea; 2 Department of Neurology, Chosun University School of Medicine, Gwangju, South Korea; 3 Department of Neurology, Research Institute of Clinical Medicine of Jeonbuk National University – Biomedical Research Institute of Jeonbuk National University Hospital, Deokjin-gu, Jeonju-si, Jeonbuk-do 54907 South Korea

**Keywords:** Lithium, Neuroleptic malignant syndrome, Reversible splenial lesion

## Abstract

**Background:**

Lithium is primarily used to treat bipolar disorder and is known to cause several acute neurological complications. Reversible splenial lesions (RSLs) may be evident in antiepileptic drug toxicity or withdrawal, infections, and other phenomena. We report two cases of RSL presenting as neuroleptic malignant syndrome-like symptoms (NMSLS) with lithium associated neurotoxicity.

**Case presentation:**

A 28-year-old woman was admitted after taking increased dosages of lithium for schizophrenia. She experienced generalized tremor, rigidity, dysarthria, high fever, and tachycardia. Symptoms and brain lesion recovered 2 weeks after discontinuation of lithium. The second case involved a 59-year-old woman who was receiving treatment for bipolar disorder since 1988. When lithium was administered for impatience and aggressive behavior, her mental state deteriorated and fever developed, along with generalized tremor in the extremities. Brain magnetic resonance imaging (MRI) in both patients showed a reversible oval-shaped lesion localized to the splenium of the corpus callosum. Both patients were defined as neuroleptic malignant syndrome-like symptoms (NMSLS) based on the DSM-5 diagnostic criteria for neuroleptic malignant syndrome. The suspected etiology of our cases was lithium associated neurotoxicity according to their clinical course and medical information. Our patients fully recovered in 10–14 days after the discontinuation of lithium.

**Conclusions:**

The patients experienced similar clinical courses and had similar radiological findings of RSL. Manifestations in both cases were related to lithium associated neurotoxicity and this should be considered in patients with RSL and NMSLS.

## Background

Lithium is often used as a treatment of choice for patients with bipolar disorders [1]. Despite its utility in psychiatric treatment, lithium has a narrow therapeutic range, which may easily lead to unexpected neurological complications such as encephalitis, convulsion, tremor, gait ataxia, altered mental state, and neuroleptic malignant syndrome-like symptoms (NMSLS) [2]. Reversible splenial lesion (RSL) of the corpus callosum has been reported to be a nonspecific finding associated with clinical symptoms of mild encephalitis or encephalopathy. Several etiologies, including antiepileptic medication, alcohol abuse, hypoglycemia, infection, electrolyte imbalance, and trauma are related to the development of RSL [3]. Although RSL has various causes, an association with psychiatric drugs, such as lithium, has not been proven. We encountered two patients with RSL and NMSLS who had taken lithium for bipolar disorder.

## Case presentation

### Case 1

**Fig. 1:**
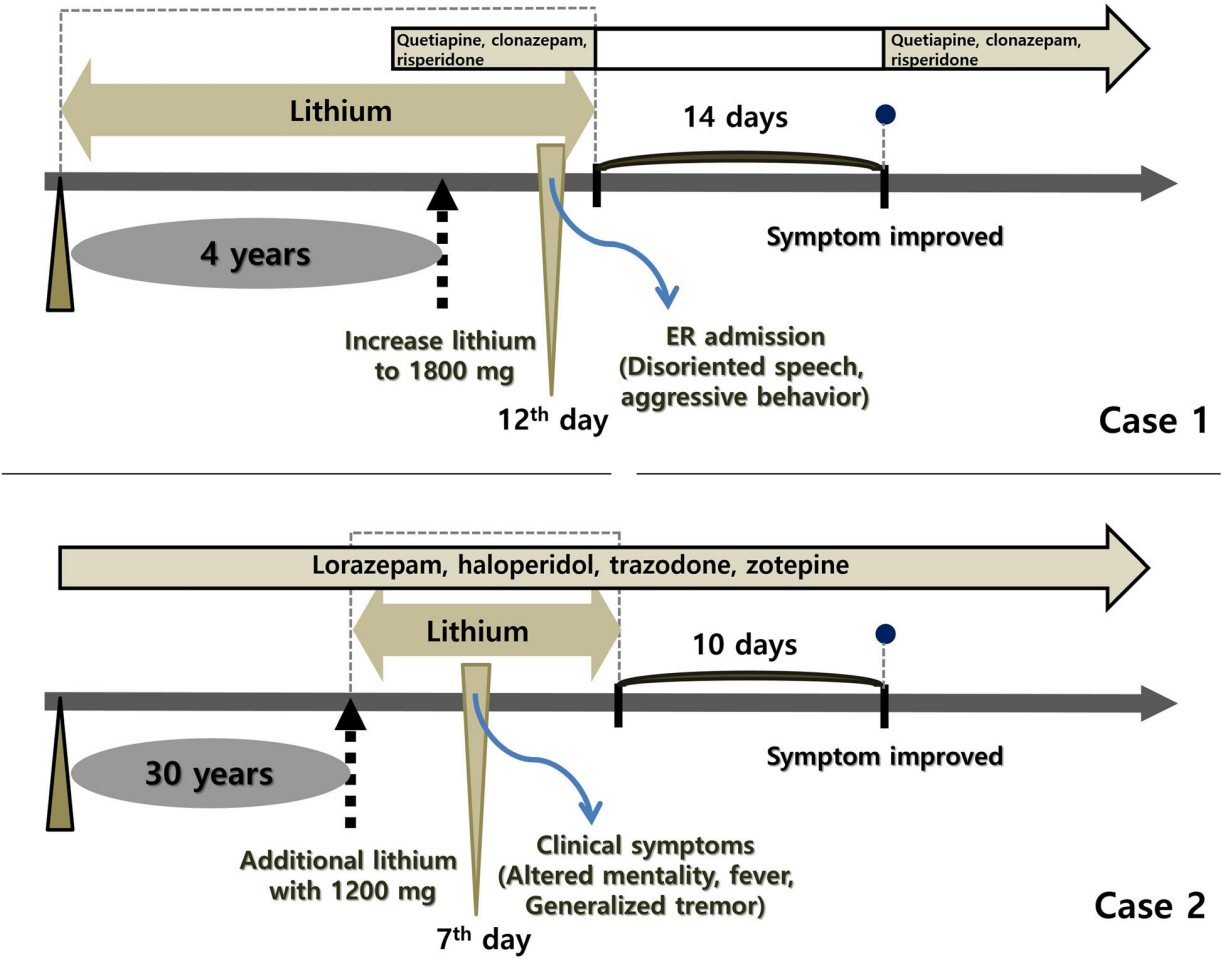
Timeline and clinical course of the patient. The neuroleptic malignant syndrome symptoms disappeared after discontinuing lithium treatment, by 14 and 10 days, respectively

**Fig. 2:**
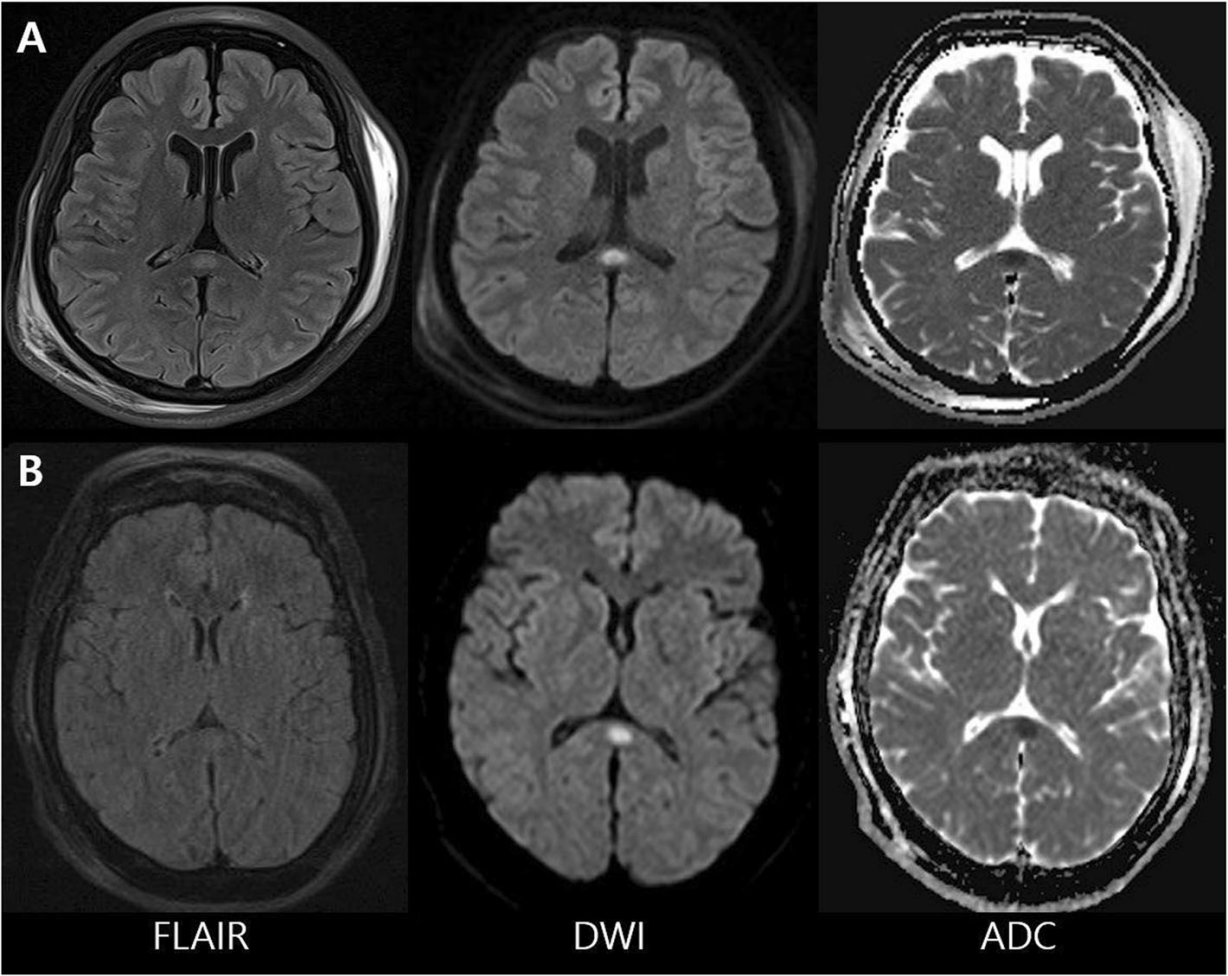
Brain magnetic resonance imaging of the two patients (**a**, Case 1; **b**, Case 2). Diffusion weighted imaging in both demonstrated a high signal intensity lesion in the mid portion of the splenial corpus callosum and showed low signal intensity on the apparent diffusion coefficient map, suggestive of cytotoxic edema

A 28-year-old woman, who had schizophrenia since 2014, was referred to the emergency department in an altered mental state. She had been taking lithium at 1200 mg/day for 4 years: quetiapine at 400 mg/day, clonazepam at 0.5 mg/day, and risperidone at 5 mg/day for several months (Fig. 1). The lithium dosage was increased to 1800 mg/day, 12 days before admission, due to her disoriented speech and aggressive behavior. The patient was referred to our hospital after 2 days of generalized tremor, rigidity, dysarthria, high fever (38 °C), and tachycardia (120 beats/min). Vital signs at admission included blood pressure at 130/100 mmHg, heart rate at 104 beats/min, and body temperature at 37.2 °C. Her lithium level was 1.24 mmol/L, slightly higher than the upper normal limit (0.5–1.0 mmol/L). The patient was diagnosed with NMSLS, and all medications, including lithium, were discontinued. Brain magnetic resonance imaging (MRI) revealed an oval-shaped, high signal intensity lesion on diffusion-weighted imaging (DWI) with decreased apparent diffusion coefficient (ADC) values localized to the splenium of the corpus callosum (Fig. 2a). The patient fully recovered in 2 weeks and experienced no relapses after further treatment with quetiapine, clonazepam, and risperidone.

### Case 2

A 59-year-old woman in an altered mental state, who was diagnosed as having bipolar disorder since 1988, was referred to the hospital due to impatience and aggressive behavior. As treatment with lorazepam 0.5 mg/day, haloperidol 5 mg/day, trazodone 25 mg/day, and zotepine 50 mg/day for 2 days was insufficient in controlling her symptoms of hyperactivity with grandiosity and decreased need for sleep. Lithium (1200 mg/day) was added (Fig. 1). Two days later, her mental capacity declined. After 7 days, she developed fever (38 °C) and generalized tremor in both the arms and legs. Her blood pressure, heart rate, and respiration rate were 150/100 mmHg, 80 beats/min, and 20 breaths/min, respectively. Laboratory investigations revealed mild leukocytosis but no other specific abnormal values. Her lithium level was 0.31 mmol/L and a cerebrospinal fluid study revealed no abnormalities. Brain MRI revealed swollen splenium of the corpus callosum and restriction in diffusion was observed on DWI with decreased ADC values (Fig. 2b). The patient fully recovered in 10 days after the discontinuation of lithium only.

## Discussion and conclusions

As our present cases had a symptoms of NMS but does not match the diagnostic criteria of NMS exactly established by DSM-5, we described our patients as NMSLS (Table 1) [4]. The clinical presentation of RSL is nonspecific and depends on the event that may have caused the condition, such as encephalopathy or encephalitis. In a previous study, patients who showed RSL by brain MRI presented with a range of symptoms including confusion, gait ataxia, and seizure [3].

**Table 1:** DSM-V Diagnostic Criteria for NMS [4]

A. Development of severe muscle rigidity and elevated temperature associated with the use of neuroleptic medication	
B. Two (or more) of the following	
1) Diaphoresis	
2) Dysphagia	
3) Tremor	
4) Incontinence	
6) Mutism	
7) Tachycardia	
8) Elevated or labile blood pressure	
9) Leukocytosis	
10) Laboratory evidence of muscle injury (eg, elevated creatine phosphokinase)	
C. The symptoms in criteria A and B are not due to another substance or a neurological or other general medical condition	
D. The symptoms in criteria A and B are not better accounted for by a mental disorder.	

The causal mechanisms of RSL are unclear. Although the splenium has a relatively weak blood supply from the posterior pericallosal artery compared with other parts of the corpus callosum, it is unlikely that lesions are caused by ischemic injury. A previous study showed that restricted diffusion imaging was reversible, with no residual lesion on follow-up with fluid attenuated inversion recovery MRI. In addition, the morphology and position of the lesion did not match those of an ischemic lesion. Several possible mechanisms have been suggested as the cause of RSL, one of which is arginine vasopressin-induced disruption of body fluid homeostasis [5]. Others have proposed reversible demyelination due to infectious diseases or anti-epileptic drug toxicities. Regardless of the etiology, splenial lesion has a tendency toward a benign clinical course and reversible radiological findings on MRI. Some researchers have proposed a new clinicoradiological syndrome for RSL [6].

The suspected etiology of RSL in our cases was lithium associated neurotoxicity, which was associated with the clinical symptoms of NMSLS. In the first case, lithium had been taken for 4 years and the dose adjustment increased the level beyond the therapeutic range. We speculate that lithium intoxication played an important role in causing RSL. Despite the normal lithium level in the second case, lithium associated neurotoxicity was considered the cause of NMSLS because the clinical symptoms were concurrent with lithium treatment. Lithium associated neurotoxicity does not always correlate with serum lithium levels [7]. Interaction with other neuroleptic drugs may have increased lithium associated neurotoxicity and clinical symptoms. We suspect that lithium intoxication played a crucial role in the development of RSL as abnormal symptoms and brain MRI findings in our cases recovered after tapering lithium.

Lithium has a narrow therapeutic range, which may easily permit neurological toxicity [7]. The target dose of lithium is 0.6–1.2 mEq/L for maintenance and 1.0–1.5 mEq/L for manic episode control; nevertheless, neurological toxicity may be evident despite being in the therapeutic range [7]. Several cases have been reported that lithium with antipsychotics might be associated with NMS [8, 9]. Those cases suggested that lithium toxicity can occur neurotoxic effect with NMS, but none of them showed combined lesion of RLS. To our best knowledge, there has been only one RSL case reportedly related to lithium intoxication; the serum lithium level was in the upper limit of the normal range (0.94 mEq/L) and NMSLS was not present [10]. In both of our cases, the initial splenial lesion on MRI had disappeared upon follow-up examination, suggesting cytotoxic edema. Reversible splenial lesion is reported to have a consistent pattern of neuroimaging abnormalities characterized by round lesions with hyperintense signals in fluid-attenuated inversion recovery MRI and hypointense signals in T1-weighted images [11].

We experienced two cases of RSL with lithium associated neurotoxicity. Therefore, careful monitoring and evaluation of patients taking antipsychotic drugs and lithium, who show toxic neuropsychiatric symptoms, is recommended. Lithium intoxication should be considered a possible etiology of RSL with NMSLS, and this approach may help to differential diagnosis of additional workup It would be important that the NMS patients systematically get an MRI to detect RSLs, especially the patients with changes in consciousness.

## Data Availability

All data and material supporting our findings are contained within the manuscript.

## Abbreviations

ADC: apparent diffusion coefficient
DWI: diffusion-weighted imaging
MRI: magnetic resonance imaging
NMSLS: neuroleptic malignant syndrome-like symptoms
RSL: reversible splenial lesion
